# Cocaine and thrombosis: a narrative systematic review of clinical and in-vivo studies

**DOI:** 10.1186/1747-597X-2-27

**Published:** 2007-09-19

**Authors:** Nat MJ Wright, Matthew Martin, Tom Goff, John Morgan, Rebecca Elworthy, Shariffe Ghoneim

**Affiliations:** 1 HealthCare Department, HMP Leeds, 2 Gloucester Terrace, Armley, Leeds, LS12 2TJ, West Yorkshire, UK; 2 School of Medicine, University of Leeds, Room 7.10, Worsley Building, University of Leeds, Leeds LS2 9JT, West Yorkshire, UK

## Abstract

**Purpose:**

To systematically review the literature pertaining to the link between cocaine and either arterial or venous thrombosis.

**Procedures:**

Narrative systematic review of Medline, CINAHL, Embase, Psycinfo and Cochrane databases supplemented by hand trawling of relevant journals and reference lists up to April 2007. In-vivo studies and those with clinical endpoints were included in the review.

**Results:**

A total of 2458 abstracts led to 186 full-text papers being retrieved. 15 met the criteria for inclusion in the review. The weight of evidence would support cocaine as a pro-thrombotic agent. There is evidence of it activating thrombotic pathways. The effect of cocaine upon clinical endpoints has not been quantified though there is evidence of an association between cocaine and myocardial infarction particularly amongst young adults. Cocaine may also be a causal agent in cerebrovascular accident though studies lacked sufficient power to determine a statistically significant effect. There is a gap in the evidence pertaining to the issue of cocaine and venous thrombosis.

**Conclusion:**

Clinicians should consider questioning for cocaine use particularly amongst young adults who present with cardiac symptoms. More epidemiological work is required to quantify the effect of cocaine upon both arterial and venous clotting mechanisms.

## Background

Globally cocaine use is common. The most recent data available for England showed a total of 147781 drug users in contact with drug treatment services and general practitioners for the current year 2007 [1]. The United Kingdom has the highest prevalence of both lifetime and recent cocaine use in Europe, and use tends to be highest in urban areas. Recent national population surveys conducted in Europe showed a lifetime prevalence of 6.8% for the UK adult population (i.e. used cocaine at least once). Levels of use among younger adults tend to be higher than the population average. The range of lifetime experience among European15- to 34-year-olds is between 1% and 11.6% and the UK is at the top of this range [2].

Cocaine is derived from leaves of the erythroxylum coca plant [3] which is grown in the Andes mountains in South America. Cocaine is available in different forms. When treated with hydrochloric acid it becomes cocaine hydrochloride salt which is water soluble and decomposes on heating. Such properties make this form amenable to intravenous injection, or snorting through the nasal mucosa [4]. In contrast cocaine alkaloid (also known as freebase or crack cocaine) is an insoluble crystalline substance that that when heated converts to a stable vapour that can be inhaled [4]. Whilst freebase and crack are the same chemical form of cocaine, they are made by different techniques. Freebase is made by dissolving cocaine hydrochloride in water then adding ammonia as a base and ether as a solvent. The cocaine base dissolves in the ether layer which is then extracted by evaporating ether at low temperatures. Cocaine freebase can then be mixed with tobacco and smoked or heated in special cocaine pipes and inhaled. Crack cocaine is made from dissolving cocaine hydrochloride in water, and then heating with baking soda. The cocaine base then precipitates into a hard mass (often known as "rocks"). This form of cocaine tends to be smoked [4], though can also be injected.

The properties of cocaine to cause vasoconstriction of the arterial vasculature have been well documented [5]. However there have been a number of case reports and series where cocaine has been implicated as the causal agent in arterial thrombosis. There are case reports of thrombosis in the renal artery [6], pulmonary artery [7], aorta [8], and coronary arteries. In some of these case reports myocardial infarction has occurred where there is no evidence of atherocsclerosis [9]. This state is often referred to in the literature as "myocardial infarction with normal coronary arteries". [10] The postulated mechanism of action is adrenergically mediated increases in myocardial oxygen consumption, vasoconstriction of large epicardial arteries or small coronary resistance vessels leading to coronary thrombosis [10]. However it has also been postulated that such infarctions could be due to a state of blood hypercoagulability leading to arterial thrombosis [10]. Hypercoagulability occurs with low plasma tissue plasminogen activator activity, high tissue plasminogen activator inhibitor activity, factor XII deficiency or abnormal platelet aggregation [10]. This raises the hypothesis as to whether in addition to properties of vasospasm cocaine is a pro-thrombotic agent.

Cocaine has also been implicated in cases of cerebral thrombosis [11] but also in cases of *haemorrhagic *cerebrovascular accidents [11]. It has been postulated that haemorrhagic cerebral infarcts in cocaine use are due to episodic hypertension due to the vasoconstricting properties of cocaine [12].

As well as being implicated as the causal agent in the process of arterial thrombosis, cocaine has also been implicated as the causal agent in venous thrombosis as it has been associated with case reports of upper extremity deep vein thrombosis [13].

The evidence from in-vitro studies is conflicting with some results showing an increase in platelet activation following cocaine administration [14] and other results showing cocaine to be an inhibitory factor in platelet coagulation (and hence thrombus formation) [15,16]. However biochemical mediators can act differently in-vitro to the human in-vivo setting. Similarly the results of animal studies have shown conflicting reports on the ability of cocaine to induce platelet formation [17,18]. Therefore this research sought to undertake a systematic review of human in-vivo studies, and studies with a clinical endpoint studying the effect of cocaine on either the arterial or venous clotting mechanisms.

## Methods

The following medical databases were searched: Medline (1966 to April 2007), EMBASE (1980 to April 2007), psycINFO (1985 to April 2007), CINAHL (1982 to April 2007), Web of Science (1981 to April 2007) and Cochrane Database to April 2007. A full copy of the search strategy is available from the authors upon request. Briefly the umbrella terms of "cocaine dependence", "thrombogenensis" and "clotting factors" were used to identify primary research relating to the topic area.

Additionally the contents pages of high impact journals were hand trawled for the period January 1999 to May 2007. The review was not limited to publications in the English language and the potential for identifying relevant grey literature material was through discussion with experts in the field. The search was undertaken by three researchers (NW, RE and MM) who independently assessed which full-text papers should be retrieved from the abstracts and reference lists. Discrepancies were resolved by discussion. Upon retrieval of the full-text papers, the names of the authors were concealed so that reviewers were blind to the author team of the papers under scrutiny.

The following inclusion criteria were applied: observational or intervention studies of participants with diagnosed cocaine abuse or dependence, or intervention studies conducted amongst human subjects administered pharmacological cocaine and evaluating any one of the following outcomes: clinical outcomes of thrombogenesis (e.g myocardial infarction, cerebrovascular accident, deep vein thrombosis), surrogate markers of raised clotting factors. As the vasoconstricting properties of cocaine are well known, studies which considered only the endpoint of vasospasm were excluded.

Additionally the following papers were excluded: editorials, discussions, opinion pieces, qualitative studies, quantitative in-vitro studies, animal studies, descriptive studies, observational studies that did not have a control group.

Selection of papers for inclusion in the review entailed independent assessment by three researchers (JM, TG and NW). Any discrepancy was resolved by a fourth independent researcher checking the papers (SG). If agreement could still not be reached then disagreement would be resolved by discussion.

The protocol for the systematic review entailed devising a checklist to assess the quality of the papers. The section of the protocol pertaining to intervention studies was informed by the recommendations of the Cochrane handbook for conducting systematic reviews[19]. Quality criteria for assessing observational cohort studies utilised established principles of research rigour in epidemiological research [20]. At the outset it was written into the protocol that negative findings of statistical significance should not be excluded. Similarly it was determined that underpowered studies whereby a statistically significant effect of an intervention could not be demonstrated were not excluded purely on the basis of lack of statistical power.

A checklist was developed to assess the quality of the studies which is available from the authors on request.

With respect to data analysis, a meta-analysis was not performed as the review protocol did not limit inclusion solely to RCTs. Rather in line with current recommended practice for analysis of non-randomised studies, a narrative analysis of the papers was adopted to elicit common themes emerging from the studies [21].

## Results

**Figure 1:**
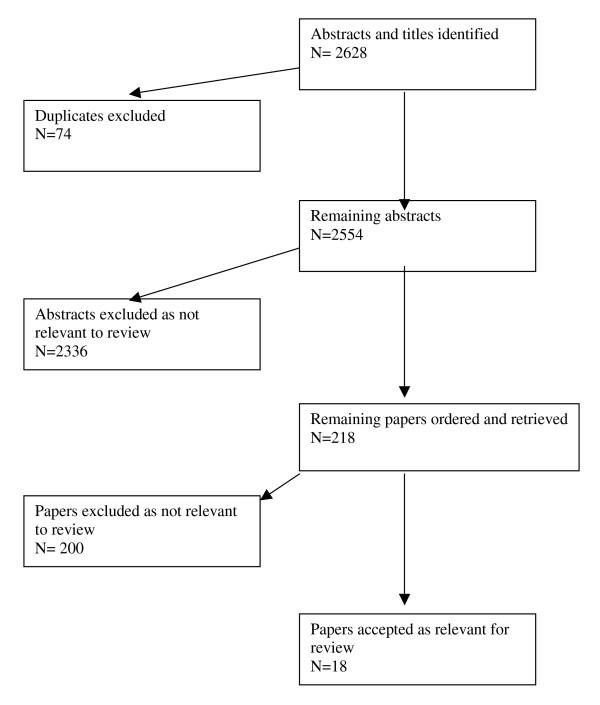
Flow chart showing process of retrieval of papers included in the systematic review.

The review found results of studies with clinical endpoints and also studies evaluating the effect of cocaine upon the surrogate markers of clotting factors. A total of 2628 abstracts led to 218 full-text papers were retrieved and 18 were included in the review (see figure 1). Summaries of the papers and their findings are shown [see Additional file 1].

### Clinical outcomes

Papers were retrieved which described the effect of cocaine upon coronary artery vasculature and cerebral arteries. Results pertaining to these clinical endpoints are described below.

### Coronary artery vasculature

Evidence for the possible thrombotic effects of cocaine comes from the study conducted by Mittleman et al in 64 medical centres in the USA [22]. Using case control cross-over methodology it showed that within 1 hour of using cocaine there was a 23.7 times increased relative risk of MI. The elevated risk rapidly decreased after one hour. This concurs with the finding by Qureshi et al that persons reporting regular cocaine use had a significantly higher likelihood of non-fatal MI than non-users (age adjusted odds ratio 6.4) [23]. It would appear that the participants with MI presented in the Quereshi et al paper were further analysed using case control methodology and presented in a further paper [24]. Results showed that compared to controls with MI, cocaine users with MI were younger with a lower number of coronary artery disease risk factors. Angigography revealed a higher level of multivessel disease (65% vs 32%, P < 0.05, confidence intervals not stated) and a higher number of coronary artery lesions (≥ 50% 2.3 per patients vs 1.6/patient, P < 0.05, confidence intervals not stated).

A case control study conducted by Tanenbaum et al demonstrated a statistically significant association between illicit cocaine use and major electrocardiograph (ECG) changes compared to schizophrenic controls [25]. Such ECG changes included myocardial infarction, myocardial ischaemia or bundle branch block. However an important limitation of this study was that cigarette smoking was more common in the cocaine using group compared to the control group and multivariate analysis to control for smoking effect was not conducted.

The findings by Dressler et al showed that autopsy coronary artery pathology was greater in those who had toxic levels of cocaine in the blood compared to those who did not [26]. They postulate that either coronary atherosclerosis is accelerated by cocaine addiction, or that cocaine provides a fatal stress in patients with premature coronary atherosclerosis from other causes. Evidence for the former comes from the finding by Amin et al showing that of those cocaine users presenting to medical departments with chest pain, there was no statistically significant difference in coronary risk factors between those who developed acute myocardial infarction and those who did not [27]. This would suggest that cocaine accelerates atherosclerosis rather than acting as a stress to those with premature atherosclerosis.

Such a process of cocaine accelerating a process of atherosclerosis is consistent with the findings from autopsy studies.

An autopsy study by Kolodgie et al 1991 reported on differences in number of mast cells in coronary artery sections plotted against degree of cross-sectional luminal narrowing [28]. It showed a positive correlation in patients with cocaine-associated sudden death and thrombosis compared to sudden death and thrombosis in those without a history of cocaine abuse. The authors concluded that mast cells with rich stores of histamine play an important part in the pathogenesis of coronary vasospasm and thrombosis, possibly by increasing lipid uptake and therefore promoting atherogenesis. A further study Kolodgie et al 1992 showed statistically significantly greater percentage of sudanophilia in thoracic aorta and abdominal aortae in those cases with a positive toxicological screen for cocaine compared to those with a toxicological screen that was negative for cocaine [29]. Sudanophilia is a marker for fatty streaks, which is an indicator of early atherosclerosis.

An autopsy study by Virmani did not reach firm conclusions [30]. Two of the cocaine cases had severe coronary atherosclerosis and one an occlusive coronary thrombus. However numbers from the control group with atherosclerosis or coronary thrombus was not stated. Whilst cocaine users showed a statistically significant increase in the histological changes of myocarditis (as shown by mononuclear infiltrate), contraction band necrosis was also less in the cocaine using group.

Whilst the findings of the autopsy studies appear to concur with those studies evaluating a clinical outcome, they do have limitations. The key limitation of the autopsy studies by Kolodgie [28,29] and by Virmani [30] is that the opportunity to calculate the relative risk of atherosclerosis in cocaine users compared to non-cocaine users was missed.

### Cerebral pathology

The paper by Fessler et al reported the findings of subarachnoid haemorrhage in those who used cocaine compared to a control group of non-cocaine using patients [31] found that compared to the non-cocaine using group, the cocaine using group had a younger age at presentation and a smaller aneurysm diameter suggesting that cocaine accelerates pre-existing pathology.

However, the Quereshi study despite recruiting 10085 participants concluded that there was no statistically significant increased risk of non-fatal stroke amongst frequent cocaine users [23]. However it would appear from the multi-variate adjusted odds ratio that despite large numbers recruited into the study it was underpowered to detect a difference (multivariate adjusted odds ratio 0.49, 95% CI 0.01–7.69). A slightly larger case control study of 10368 women however found an elevated risk of stroke in users of cocaine (Adjusted odds ratio for any cocaine 13.9, 95%CI: 2.8–69.4) [32]. The study sought to quantify the associations between stimulants and either ischaemic or haemorrhagic stroke. However the researchers aggregated data for cocaine and amphetamine use making interpretation difficult. Research aggregating data for both cocaine and amphetamine was also published by Kaku & Lowenstein(1990) [33] which showed a temporal relationship between stimulant use (either cocaine or amphetamine). The closer the time to last cocaine use, the greater the strength of association with stroke.

Therefore the current body of evidence would appear to support an association between stimulants and cerebrovascular accident with the most significant stimulant risk factor being cocaine. However more research is need to quantify the effect of cocaine upon the risk of thrombotic rather than haemorrhagic cerebrovascular accident.

### Clotting factors

The study by Heesch et al 2000 entailed administering pharmacological cocaine at a dose of 2 mg/kg to healthy volunteers. Relative to placebo administration there was a statistically significant increase in platelet factor 4, β-thromboglobulin clotting factors. There was also an increase in platelet containing microaggregate formation and a reduction in bleeding time [34]. This finding appears to be more significant than the findings by the same author reported in 1996 [35]. In this study whilst cocaine was administered in-vivo, platelet aggregation was induced in-vitro using either collagen, adenosine phosphate epinephrine, or arachadonic acid. There was a trend towards decreased aggregation regardless of which drug was used. However following adenosine phosphate administration there was a statistically significant greater reduction in platelet aggregation in the cocaine group compared to the placebo group.

A consistent theme emerges from the results of other in-vivo studies of cocaine leading to an increase in clotting factors. A study by Rinder et al aggregated cross-sectional data from an observational study and baseline data from a pilot controlled clinical trial [36]. The results were a higher resting level of P-selectin positive platelets in cocaine users compared to healthy controls which the authors concluded could mediate a process of thrombosis.

The study by Moliterno et al demonstrated a statistically significant increase in plasminogen activator inhibitor (PAI-1) after cocaine administration[37]. Elevated levels of PAI-1 are associated with thrombogenesis. The biochemical pathway for thrombolysis is such that tissue plasminogen activator coverts plaminogen to plasmin which in turn causes fibrinolysis by degrading fibrinogen and fibrin clots. PAI-1 inactivates tissue plaminogen activator. The paper by Siegel et al 1999[38] showed an increase in von Willebrand factor, haemoglobin, haemotocrit and red cell count that was dose related to intravenous cocaine administration. Changes were observed at a dose of 0.4 mg/kg cocaine but not at a dose of 0.2 mg/kg. The authors concluded that cocaine induced a transient erythrocytosis that may increase blood viscosity. They also concluded that an increase in von Willebrand factor without a compensatory change in endogenous fibrinolysis may trigger platelet adhesion, aggregation, and intravascular thrombosis. A further study by Siegel et al reported in 2002 used case-control methodology to compare clotting factors in cocaine dependent users versus those who abused but were not dependent on cocaine [39]. Those with dependant cocaine usage showed elevations in C-reactive protein, von Willebrand factor and fibrinogen. Fibrinolytic activity and total cholesterol showed no difference between the 2 groups. The authors concluded that the findings were consistent with a cocaine-related inflammatory response with pro-thrombotic effects.

## Discussion

The weight of evidence from both clinical and in-vivo studies included in this review would suggest that cocaine has pro-thrombotic properties and can be responsible for early-age onset of cardiovascular morbidity and mortality. It could be argued that observational studies with clinical endpoints and in-vivo studies are too heterogenous to allow firm conclusions to be drawn as the former considered the effect of exposure to illicit "street" cocaine, whereas the latter considered the effect of pharmacological cocaine. However we felt that having a search strategy that limited the review to just to one of either clinical or in-vivo studies would have been over-focussed and risk missing important data. There is strong evidence for cocaine being a risk factor for myocardial infarction and moderately strong evidence for cocaine being a risk factor for cerebrovascular accident.

Further the conclusions drawn from our review concur with findings from the wider literature suggesting that cocaine has a cardiotoxic effect that is not just limited to thrombogenensis. The findings included in this review by Mittleman et al of an elevated risk of MI one hour after cocaine use concurs with a US based prospective observational study of 246 participants [22]. This study examined the characteristics of cocaine associated chest pain and found that pain occurred a median of 60 minutes after cocaine use and persisted for up to 120 minutes [40]. A case control study of 50 patients, whilst not considering directly the outcome of thrombogenesis did demonstrate an association between cocaine use and the presence of contraction bands in the myocardium (which may act as the anatomic substrate for arrhythmias associated with cocaine use)[41]. A controlled clinical trial evaluating the effect of cocaine on ECG and echocardiographic changes before and after a single intravenous dose of high dose cocaine showed a doubling in the frequency of hyperdynamic left ventricular wall segments after high dose cocaine compared to placebo and dose related non-specific changes on ECG [42]. These findings concur with ECG and echocardiographic study findings conducted on 52 *chronic *cocaine abusers.

Compared to controls, chronic cocaine abusers had increased left ventricular posterior wall thickness, increased septal wall thickness and higher left ventricular mass index [43]. A controlled trial of 18 patients undergoing cardiac catheterisation for evaluation of chest pain found that post administration of cocaine, the magnitude of vasoconstriction was greater in diseased areas (of atherocsclerosis) compared to non-diseased areas[44]. Our findings would suggest that recent cocaine use should be routinely inquired of all young patients attending health services with symptoms of chest pain. A USA based study of 359 patients presenting to emergency departments with chest pain of possible cardiac origin revealed 17% had cocaine or cocaine metabolites in the urine [45]. They tended to be younger than those without cocaine metabolites in the urine. A USA based retrospective cohort study of cocaine associated myocardial infarction revealed a low post MI mortality and the majority of complications occurring within 12 hours of presentation [46]. It is possible that the relative young age of those with cocaine associated MI protects against further complications.

The clinical management of those with cocaine induced cardiovascular pathology has been discussed in the literature. Commentators practicing in the area of emergency medicine have argued that those with suspected acute cocaine induced myocardial ischaemia or infarction should be treated similarly to those with acute coronary syndromes but with some exceptions [47]. These include administering benzodiazepines in the early management to reduce the central nervous stimulatory effects of cocaine; avoiding B blocker medication as it may exacerbate cocaine induced coronary artery vasoconstriction; and a preference for percutaneous coronary intervention over fibrinolysis as cocaine associated chest pain in young cocaine users is associated with low mortality. Also many patients are hypertensive and aortic dissection must be considered. Therefore on balance the risk/benefit ratio would not favour administration of fibrinolytics.

As regards primary prevention of cocaine induced cardiovascular morbidity, we could only find one study which evaluated the effectiveness of aspirin. The study showed that aspirin is ineffective at protecting against platelet aggregation and cerebral hypoperfusion in cocaine users. Rather abstinence from cocaine was shown to be effective in reducing platelet abnormalities and increasing cerebral perfusion [48].

Our findings raise further implications for research. There is a need to quantify the absolute risk of circulatory morbidity and mortality due to illicit cocaine use by utilising longitudinal observational methodology with a control group of non-cocaine users. Additionally more research is needed to further understand the effect of cocaine upon cerebral artery vasculature. The evidence for cocaine as a causal agent in cases of cerebrovascular accident is not conclusive. However the findings of this review concur with the wider evidence base. An early descriptive paper which reviewed the records of 3712 drug abusers and highlighted 13 patients with cerebrovascular accident, of these 7 were ischaemic in nature and the mean age was 34.2 years [49]. Such descriptive data would lend support to more rigorous epidemiological work quantifying the relative risk at developing an ischaemic cerebrovascular accident following cocaine use.

Our review did not highlight any papers studying the effect of cocaine upon venous vasculature. There are descriptive studies hypothesising cocaine as the causal agent in deep vein thrombosis [13]. It has been hypothesised that it could be either cocaine itself or an adulterant used to dissolve cocaine prior to injection in the bloodstream that is the putative agent for thrombus formation [13]. This area merits further in-depth research with more specific questioning regarding the form and strength of cocaine that is used for injection.

The effect of different forms of cocaine upon the circulatory system also merits further activity. We retrieved one paper in which the authors had aggregated data from case reports in the literature with their own case series [50]. They concluded that ischaemic and haemorrhogic strokes were equally likely after taking alkaloidal (crack) form of cocaine, whereas the cocaine hydrochloride form is more commonly associated with haemorrhagic stroke. However any attempt to control for confounders was not mentioned. This coupled with the fact that positive reporting bias could not be excluded from the case reports which informed the aggregate data means that firm conclusions could not be drawn from the data. Similarly the findings by Petitti et al [32] whilst adjusting for confounders only presented adjusted odds ratios for risk of haemorrhagic or ischaemic stroke in users of "cocaine and/or amphetamine" which limits firm conclusions regarding the effect of type of cocaine upon thrombogenensis.

## Conclusion

In summary the weight of evidence would support the hypothesis that cocaine is thrombogenic though more rigorous observational research with clinical endpoints is needed to quantify relative risk. From our findings we would suggest that where young people present with symptoms consistent with acute cardiovascular events, direct questioning should include all relevant drugs. As regards primary prevention advice and support to become abstinent from cocaine is more likely to improve health outcomes than the provision of prophylactic aspirin.

## Competing interests

The author(s) declare that they have no competing interests.

## Authors' contributions

NW had the original idea for the research, devised the search strategy, read and reviewed the papers independently from the other reviewers and wrote the first draft

MM, RE ran the search and retrieved the papers. TG, JM independently read the papers against the inclusion/exclusion criteria. SG – resolved any discrepancy by independently checking the papers that had been recommended for inclusion/exclusion by the initial reviewers, edited the final draft of the manuscript, and managed the references. All the authors read and approved the final manuscript.

## Supplementary Material

Additional file 1 Table 1: Clinical and in-vivo studies included in the systematic review. Collated data of all accepted papers for this review. Click here for file
