# Supplementary material for: Cocaine and thrombosis: a narrative systematic review of clinical and in-vivo studies
Source: Subst Abuse Treat Prev Policy. 2007 Sep 19;2:27. doi: 10.1186/1747-597X-2-27 (PMC2042971; doi:10.1186/1747-597X-2-27)
Supplement: Additional file 1 — Table 1: Clinical and in-vivo studies included in the systematic review. Collated data of all accepted papers for this review. [file 1747-597X-2-27-S1.doc]

**Table 1: Clinical and in-vivo studies included in the systematic review**

| **Author, year of publication and dates of recruitment** | **Setting** | **Participants (number, age, gender, ethnicity)** | **Methods (including inclusion/exclusion criteria and quality of methodology)** | **Intervention** | **Outcomes** | **Results** |
| --- | --- | --- | --- | --- | --- | --- |
| Amin et al 1990(27)  Recruited – 1985-1989 | Montefiore Medical Centre, Bronx, New York | 70 cases of chest pain and cocaine (mean age 34 +/- 9, 56 men, 14 women, ethnicity not stated. 22 had acute MI (mean age 36 +/- 10 years, 21 men, 1 woman)  48 had not had an acute MI (mean age 33 +/- 9, 35 men, 13 women) | Retrospective Cohort Study. A list patients admitted with a history of chest pain and cocaine use was generated using ICD-9 codes. And a second list generated by cross referencing MI and coronary artery disease with cocaine use.  75 admissions but only 70 charts available for review.  25 smoked crack cocaine; 24 used intranasal cocaine; 8 used IV cocaine; 13 route of administration wasn't recorded  No statistically significant difference in the incidence of risk factors between those who developed (AMI) and those who did not (supporting stats not provided). | N/A. Observational Study | Recording on medical charts of ECG changes, peak creatine kinase, creatine kinase MB fraction, stress test, and cardiac catheterisation results conducted immediately post-admission. | Acute myocardial infarction (AMI) developed in 22 (31%) and transient myocardial ischaemia in an additional 9 patients (13%). The presenting ECG was abnormal in 20 of the 22 AMI's and in 19 of 48 of the non AMI's. Mean creatine kinase levels 1249 +/- 1520 IU/litre in the AMI group and 243 +/- 296 IU/litre in the non-AMI group (P<0.001). Creatine kinase MB elevations were only observed in the AMI group. AMI was not associated with route of cocaine administration (supporting stats not provided). No patient had >2 coronary risk factors. |
| Dressler et al 1990(26)  Recruited – 1979-1989 | Pathology Department, Maryland, USA | 22 patients studied at necropsy. Age range 15-50. Gender -17 men, 5 women. Ethnicity 19 black, 3 white)  13 had deaths associated with increased cocaine levels. | Necropsy of 22 known cocaine addicts, who were also known not to use opiates.Divided into two groups. Group 1 - cocaine related death (13 patients; mean total blood cocaine level 0.36mg/dl).  Group 2 - non-cocaine related deaths (9 patients with normal total blood cocaine level). Each heart was initially examined, 18 of which were then re-examined. | N/A. Observational study | The four major coronary arteries (left main, left anterior descending, left circumflex, and right) were examined for signs of atherosclerosis and recorded as extent of narrowing in cross-section (0-25%, 26-50%, 51-75%, 76-95%, 96-100%) | 6/13 of cocaine related death group had 1 or more major coronary arteries severely (>75% of cross-sectional area) narrowed by atherosclerotic plaque) cf 2/9 non-cocaine related death group. (comparative statistics not reported) |
| Fessler et al 1977(31)  Recruited - 1988-1993 | Detroit Medical Centre, Michigan, USA | 33 patients presented with neurovascular sequelae associated with cocaine use. Age range 24-68, mean age 37.2, 17 male, 16 female, ethnicity not stated.  44 Control patients. Mean age 52.2 years, other demographics not stated) | Case control study.  Cases included: self report chronic cocaine use (30 used crack cocaine, 3 used cocaine hydrochloride nasally), confirmed either by urine sample or report of family member/friend and presenting with neurovascular symptoms (87.9% reported symptoms within 6 hours of using cocaine) and neurovascular sequelae. Control group included non-cocaine using patients presenting with aneurysmal subarachnoid haemorrhage (SAH) | N/A. Observational  study | CT scan of the brain to detect (SAH) or intracerebral haemorrhage (ICH) secondary to cerebral aneurysm conducted in the emergency room within 2 hours of presentation.. | CT scan was performed in 31/33 cases.16 patients presented with SAH, 6 presented with ICH, and 7 with ischaemic stroke. The average age of patients who used cocaine and presented with SAH secondary to a ruptured intracerebral aneurysm was 32.8 years with an average aneurysm diameter of 4.9mm versus an average age of 52.2 years and an average aneurysm diameter of 11.0mm in non-cocaine users. (significant to p<0.05, confidence intervals not stated) |
| Heesch, 2000 (34) Recruited – Not stated | Laboratory Study, Pittsburgh , USA | 14 healthy volunteers (10men, 4 women) aged 23-41 yrs. Ethnicity not stated. | Randomised double blind crossover trial  All denied history of drug abuse and all non-smokers. No therapeutic drugs or other agents taken for at least 2 wks before each study day. Negative urine pregnancy tests obtained from all women the day before each study. | Each subject received cocaine 2 mg/kg once and a similar volume of saline placebo once, in random order by intranasal administration. Washout period of at least 2 wks between the study days. | Baseline bleeding time measurement done on R.forearm, parallel to the long axis of the arm, using a Simplate device. 30 mins later, baseline blood sample obtained for determination of platelet activity. Intervention then given. Blood sampling repeated 40, 80 and 120 mins . 2^nd^ bleeding time determination done at end of the study. † | Increase in both platelet factor 4 [mean (SD), 16 (7) to 39 (22) IU/ml, p=0.04] and β thromboglobulin [70 (20) to 98 (26) IU/ml, p<0.01] at 120 minutes following cocaine administration. Platelet containing microaggregate formation was increased at 40 minutes [from 47 (3.2) % to 54 (2.0) %, p<0.001] and 80 mins [55 (2.5) %, p=0.04]. Bleeding time decreased following cocaine [from 10 (1) to 9 (1) mins (p=0.07)]. No changes in any of the measured variables were noted following placebo administration. |
| Heesch et al, 1996 (35) , Recruited – Not stated | Setting not stated Dallas, USA | 12 healthy male volunteers aged between 23-46 yrs. Ethnicity not stated. | Double blind randomised controlled crossover trial,washout period of at least 2 weeks between studies. Exclusion criteria: smoking, history of drug abuse, or taking medications (including over the counter medications) in the two weeks preceding the study | Each volunteer received cocaine, 2mg/kg (as a 10% cocaine HCl solution), or a similar volume of saline solution by intranasal administration in random order | At baseline, 40, 80 and 120 mins after administration of the study drug blood samples obtained. At each time point platelet aggregation was induced in vitro in 4 specimens using 0.5, 1, 2 and 5 μg/mL collagen, in 4 specimens using adenosine phosphate (ADP) (1, 2, 4, and 10 μM), in 3 specimens with epinephrine (10,25, and 50 μM) and in 1 specimen with 625 μM arachidonic acid. Aggregation testing was maintained for 4 mins after induction with collagen and ADP, and for 12 mins after induction with epinephrine and arachidonic acid. For the two low concentrations of collagen and ADP, and for arachidonic acid specimen, peak aggregation of the curves was determined (as with these agents at these concentrations most curves proceed to a peak value of primary aggregation followed by disaggregation. For the two high concentrations of both collagen and ADP, and for all epinephrine concentrations, aggregation measuered at end of testing period. | No difference in aggregation at any agonist concentration used between the placebo and cocaine group at baseline. Trend toward decreased aggregation over time regardless of which study drug was given, which reached statistical significance (p<0.004) when aggregation was tested with collagen (1 μg/ml) and ADP (2 μM). Trend toward decreased aggregation with cocaine cf placebo when aggregation induced with ADP (10 μM). Decrease in aggregation after administering cocaine (as evidenced by differential time effect for the study drug variable, as represented by Study drug x time interaction, P=0.038) |
| Kaku et al 1990 (33)  Recruited retrospectively for time period Jan 1979-Dec 1988 | San Francisco General Hospital, USA | 428 participants (214 in each of the case and control group), mean age 35.4 (range 17-45), 282 men and 146 women, ethnicity not stated | Case control study.  Inclusion criteria as cases: admitted to the hospital with a diagnosis of either acute ischaemic or haemorrhagic stroke as identified through the computerised admission register  Inclusion criteria as controls: acute medical or surgical admissions for conditions for which drug abuse is not a risk factor (asthma, appendicitis and cholecystitis) matched to cases for sex, age (to within 3 years) and year of discharge matched | N/A. Observational  study | Risk ratios for drug abuse predisposing to stroke when taken within 6 hours, between 6 hours and 2 weeks, and between 2 weeks and 1 year of the acute stroke event. Logistic regression to control for confounders of cardiac disease, diabetes mellitus, smoking, hypertension, alcohol abuse and pregnancy | Risk ratios (after excluding 13 patients with strokes related to infectious endocarditis) 46.5 (95% CI:6.1-354.0) for drug abuse <6hours of stroke symptoms; 1.8 (95% CI:0.7-5.1) for drug abuse >6hours <2weeks of stroke symptoms; 0.7 (95% CI:0.0-13.7) for drug abuse >2 weeks <1 year of stroke symptoms; 5.0 (95% CI:1.4-18.1) for time period of use unknown.  Cocaine (57%) was drug used most frequently and (30%) abused more than one drug. Of the 34 patients who had stroke in temporal proximity to drug use, 31 had used either cocaine or amphetamine |
| Kolodgie et al (29) , 1992 Recruited – 1987- 1990 | University of Maryland, USA | 52 subjects identified from a larger study [number not stated: the Pathological Determinants of Atherosclerosis in Youth Study (PDAY)]. Age range 15-34 years, Ethnicity: black | Case control study. Inclusion Criteria: Black ethnic background and death due to homicide, suicide, accidents or drug overdose who were autopsied within 24 hours of death. 27 cases cocaine positive as identified by thin layer chromatography analysis of bile and urine for cocaine and its major metabolites (ecgonine methyl ester and benzoylecgonine). Positive results confirmed by enzyme immunoassay (EMIT); cocaine in the blood quantitated by gas chromatography and mass spectrophotometry.  25 controls negative for cocaine on toxiclological analysis (confirmed by police records).  Exclusion criteria: Race other than black ;congenital heart disease; Down’s syndrome; AIDS or hepatitis. 11 cases and 15 controls excluded from statistical analysis due to lack of risk factor data [total cholesterol, HDL, and smoking (differences for demographic variables between those included in the analysis and those excluded not stated)]. | N/A. Observational | Serum total cholesterol; high density lipoprotein cholesterol (HDL-C); non-HDL-C concentration [referred to as the very low density plus low density cholesterol concentration (VLDL + LDL-C)]; serum thiocyanate analysis; aortic and right coronary artery sudanophilia levels | No statistically significant difference for lipid and lipoprotein levels between case and control groups. Statistically significantly greater % sudanophilia in thoracic aorta (cocaine 28+/- 3 vs control 10+/-4; P=0.002) abdominal aorta (cocaine 22+/-3 vs control 12+/-4; P=0.049) but not right coronary aorta (cocaine 7+/- 4 vs control 4+/-5; P=0.569) |
| Kolodgie et al,(28) June 1991  Recruited - 1987-1988 | Office of the Chief Medical Examiner of the State of Maryland and Northern Virginia, USA | 5,871 autopsy reports identified, of which 495 persons tested positive for cocaine, of these 6 had total thrombotic occlusion (5male, 1female), compared with 6 randomly selected age and gender matched subjects who died from cocaine overdose (5male/1female), and 6 subjects who died suddenly from severe coronary atherosclerosis and thrombosis with negative toxicology screen for drug abuse (6M). Age range 21 to 38. Ethnicity not stated. | Case-control study  (6 cases, compared to 12 controls) | N/A. Observational | coronary arteries for atherosclerosis and thrombosis; presence of acute or healed myocardium; light microscopy of myocardium with grossly suspected pathologic change).  Coronary artery histology to evaluate extent of artery luminal narrowing by artherosclerotic plaque and presence of thrombosis; average number of mast cells per section.* | Severe coronary atherosclerosis in the 6 cases with cocaine associated thrombosis (though statistical significance not reported). Positive correlation for cocaine abuse between no. mast cells plotted against the degree of of cross-sectional luminal narrowing in the left anterior descending coronary artery (cocaine abusers r= 0.68 cf non-abusers r=0.34; P<0.03) |
| Mittleman et al 1999 (22).  Recruited – 1989-1996 | 64 medical centres, USA | 3946 patients. 2664 men and 1282 women. Age range 20-92 years.  63% (n=24) of cocaine users member of a “minority group” and 11% (n=430) of non-cocaine users member of a “minority group”. | Case control crossover study. Presenting with acute myocardial infarction. Confounders accounted for by a crossover design. Participants stratified for: no cocaine use, cocaine use in the year preceding admission, and cocaine use in the “hazard” period (i.e. the 1-hour period immediately preceding the onset of myocardial infarction symptoms). Interviewed a median 4 days after myocardial infarction | N/A. Observational | Change in MI risk due to cocaine use in the “hazard” period (the 1-hour period immediately preceding the onset of myocardial infarction symptoms) | Within 1 hour of using cocaine 23.7 (95% CI 8.5 -66.3) times increase in relative risk of MI |
| Moliterno et al, 1994 (37) , Recruited – Not stated | Cardiac Catheterization Laboratory, Dallas, USA | 22 Patients, age range 32-62 yrs, 8male, 14female. Ethnicity not stated. | RCT. Patients undergoing elective cardiac catheterisation for the evaluation of chest pain.  Patients with history of cocaine or other illicit drug use, left main coronary artery disease (as diagnosed by cineangiography of left coronary artery with a Judkins catheter), unstable angina, recent myocardial infarction, congestive heart failure, or other acute medical illness were excluded. | Baseline blood sample procured from arterial sheath, after which each subject randomly assigned to intranasal saline (group I, n=8, controls) or 10% cocaine hydrochloride, 2mg/kg (group II, n=14). 15 mins later another blood sample procured and an additional sample collected for measurement of cocaine concentration. | fibrinogen, plasminogen, tissue plasminogen activator (tPA) activity, plasminogen activator inhibitor (PAI-1) activity, and lipoprotein(a) [Lp(a)] 15 minutes after injection. † | Plasma PAI-1 activity increased after cocaine administration [0.48+ 0.06 (mean+ SD) nmol/L at baseline, 0.53+ 0.05 nmol/L after cocaine, P=0.011]. Of the plasma constituents assayed, none was altered by saline. |
| Patrizi et al 2006 (24)  Recruitment period not stated | Department of Cardiology, Rome, Italy | 51 participants. 49 men, 2 women. All Caucasian. Mean age of cases 38.8 years and mean age of controls 43.9 years | Case control study | N/A observational | Coronary risk factors and acute cardiovascular risk profile as determined by Thrombolysis in Myocardial Infarction Risk Score (TRS).  Coronary atherosclerosis as determined by coronary angiography | Compared to controls with MI, cocaine users with MI were younger with a lower number of coronary artery disease risk factors (1.4/patient vs 2.1 per patient, P<0.05). Angigography revealed a higher level of multivessel disease (65% vs 32%, P < 0.05, confidence intervals not stated) and a higher number of coronary artery lesions (≥50% 2.3 per patients vs 1.6/patient, P < 0.05, confidence intervals not stated). |
| Petitti et al. 1998 (32)  Recruitment Period. May 1991-August 1994 | Members of the Kaiser Permanente Medical Programs of North and South California, USA | 10368 women (347 cases and 1021 controls) aged 15-44 years. 55.6% white, 7.4% Hispanic, 25.9% African-american, 3.7% asian, 7.4% other/unknown | Case control study | N/A observational | History of stroke as identified by hospital admission records, hospital discharge records, emergency department logs and requests for payment for out-of-plan hospitalisations | Elevated risk of stroke in users of cocaine. Adjusted odds ratio for any cocaine 13.9 (95%CI: 2.8-69.4), for cocaine powder or paste 19.7 (95%CI: 2.3-166.3), for smoked crack cocaine 11.2 (95% CI: 1.1-118.8).  AOR for haemorrhagic stroke and cocaine and/or amphetamine 9.6 (95% CI: 2.7-33.5), for ischaemic stroke 4.5 (95% CI 0.9-21.6) |
| Quereshi et al 2001(23)  Recruited - 1988-1994 | Household survey conducted across 81 counties in USA | 10085 Participants aged 18 to 45 classified according to life-time use of cocaine:  8822 Cocaine non-users (mean age 30.8 +/- 8.0, 3908 men, 4914 women; ethnicity: 5474 white, 2961 black, 387 “other” ethnic background)  731 Infrequent users [(1-10 times) mean age 30.6 +/- 6.7; 430 men, 301 women; ethnicity: 487 white, 222 black, 22 “other” ethnic background]  532 Frequent cocaine users [(either 10-100 times or >100 times), mean age 32.8 +/- 6.5; 355 men, 177 women; 275 white, 242 black, 15 other ethnic background) | Case control study.  Black and Mexican populations, children and elderly were proportionally over sampled to obtain statistically reliable results. Participants <17 or >45 were excluded from the study. | Observational | Diagnosis given by a physician of non-fatal stroke or MI. | 46 suffered non-fatal MI's (0.5%; 39 non-users, 6 frequent users and 1 infrequent user) and 33 non-fatal strokes (0.3%; 31 non-users, 1 frequent user and 1 infrequent user). Persons reporting regular cocaine use had a significantly higher likelihood of non-fatal MI than non-users (age adjusted odds ratio 6.4, 95% confidence interval 1.25-53; multivariate adjusted odds ratio 6.9, 95% CI 1.3-58)- population attributable risk of frequent cocaine use calculated as 25%. No statistically significant increased risk of non-fatal stroke amongst frequent users (AOR 0.7, 95% CI 0.01-10.5; multivariate adjusted odds ratio 0.49, 95% CI 0.01-7.69) |
| Rinder et al, 1994 (36) Recruited – Not stated | Yale University School of Medicine, New Haven | Part A  Patient studies - 8 subjects: age, gender, and ethnicity not stated and 85 age matched controls  Part B  4 subjects age, gender, and ethnicity not stated. | Observational study (Part A) inclusion criteria were frequent users of freebase cocaine with recent abstinence.  Pilot controlled clinical trial (Part B) inclusion criteria were 2 cases with a history of cocaine use and 2 healthy volunteers | Double blind Intravenous injection over five minutes of cocaine 0.25mg/kg, cocaine 0.5mg/kg or placebo (5% dextrose in water) (Part B) | Percent of circulating activated platelets in whole blood (those that express the alpha granule membrane protein P-selectin) at baseline (part A of the study) and after 30 and 60 minutes (for part B of the sudy) | Higher resting level of P-selectin positive platelets in cocaine users (8 subjects from part A and 4 from part B) cf 85 healthy controls [11.8+/-14.4% (mean +/-SD) cf 4.4+/-3.7% (mean +/-SD); P=0.01, range 1-14%]  Post infusion of cocaine or placebo no statistically significant difference in peak circulating platelet levels (72+/-31% for 0.25mg/kg cocaine; 59+/-26% for 0.5mg/kg cocaine; 77+/-31% placebo, P>0.1) |
| Siegel et al, 2002 (39), Recruited – Not stated | Department of Medicine, and Alcohol and Drug Abuse Research Center, Mclean Hospital, Belmont, USA | 10 subjects (mean age 26+/- 5yrs) reporting 2-6 intranasal uses of cocaine/month over prior yr met criteria for abuse as defined by DSM-IV and 10 subjects (mean age 41+/- yrs) reporting 6-20 uses of cocaine inhalation per week over the year prior to recruitment and met criteria for cocaine dependence as defined by DSM-IV. Gender and ethnicity not stated | Case-control study. Participants matched for body mass index. Only confounder of cholesterol accounted for (no statistically significant difference between the 2 groups).  Those with active medical or unstable psychiatric conditions were excluded (as diagnosed from comprehensive medical or mental health examinations, electrocardiographic and laboratory testing) | N/A. Observational | C-reactive protein (CRP), von Willebrand Factor (vWF), fibrinogen and fibrinolytic activity and total cholesterol | Compared to normal values in drug-free subjects with cocaine abuse, subjects with dependant cocaine usage showed elevations in CRP (4587+/-1346 vs. 809+/-227 ng/ml, p=0.02; normal <2000 ng/ml), vWF (146.0+/-14.9% vs. 88.6 +/-14.2%, p=0.01; normal=50-150%), and fibrinogen (334.2+/-20 vs. 234+/-12 mg/dl, p=0.0004; normal=150-300 mg/dl). Fibrinolytic activity and total cholesterol showed no difference between the 2 groups. |
| Siegel et al, 1999 (38), Recruited – Not stated | Department of Medicine, Alcohol and Drug Abuse Research Center, Brain Imaging Center, and Pharmacy Department, Mclean Hospital, Belmont, USA | 21 (though numbers not explicitly stated) subjects aged 21-35 yrs. Gender and ethnicity not stated | Double-blind controlled clinical trial. Inclusion criteria: met criteria for long-term cocaine abuse as described in the DSM-IV. | Cocaine hydrochloride either intranasal at a dose of 0.9 mg/kg using a modified snort-stick device, or intravenous at doses of 0.2 or 0.4 mg/kg in a 1-mL bolus for 1 minute, or saline placebo | Hematologic parameters measured at baseline, 10, 20, 30, 40, and 60 mins and additionally at 2, 4, 8, 12, 16, 20, 120, 180, and 240 mins for plasma cocaine levels. Hemostatic factors (vWF, fibrinolytic activity, fibrinogen, plasminogen activator inhibitor antigen, and tissue-type plasminogen activator antigen) assayed at baseline 30, 60, 120, 180, and 240 mins after iv cocaine. Cardiac troponin subunits T(cTnT) and I(cTnI) sequentially measured to assess silent injury to myocardium. | Hemoglobin level (P=0.002), hemocrit (P=0.01), and red blood cell counts (P=0.04) significantly increased from 4% to 6% over baseline from 10-30 mins after intranasal (n=14) and iv (n=7) cocaine administration in doses of 0.9 mg/kg and 0.4 mg/kg, respectively (P values supported by tabular presentation of standard error of the mean data). No change in white blood cell or platelet counts. There was a significant increase (P=0.03) in vWF from 30-240 mins, peaking at 40% over baseline following iv cocaine administration in a dose of 0.4 mg/kg (n=12), with no change after 0.2 mg/kg (n=3) or placebo (n=6). Other hemostatic factors, creatinine, blood urea nitrogen, and cardiac troponin subunits T and I showed no changes. |
| Tanenbaum, 1992 (25) Recruited – Not stated | 104-bed inpatient psychiatric division of an urban, nonmunicipal academic hospital center New York | 70 men and 29 women, age 32 +/- 7yrs (mean +/- SD), who met DSM III Axis Icriteria for cocaine abuse. 31 men and 19 women, mean age 32 +/- 7yrs (mean +/- SD) patients who met who met DSM III Axis Icriteria for schizophrenia. 43% cocaine-abusing group Caucasian, 64% schizophrenic controls Caucasian | Case-control study.  Confounders not accounted for in analysis (smoking was significantly more common amongst cocaine users vs controls – 76% vs 36% X^2^ = 16.13, P< 0.001)  Ethnicity difference was statistically significant (X^2^ = 4.83, P< 0.05, df=1) | N/A. Observational | Significant ECG findings defined as evidence of myocardial infarction, myocardial ischemia, or complete bundle-branch block (RBBB or LBBB). Excluded were sinus arrhythmias, intraventricular conduction delays, non-specific ST-T wave changes, and atrial or ventricular hypertrophy | 11/ 99 patients in cocaine-abusing group had major ECG findings, 0/50 of schizophrenic controls had major ECG findings (X^2^=4.48, df=1, p<0.05). 6 patients had ECG findings of myocardial infarction, 3 had myocardial ischemia, 1 had myocardial infarct combined with RBBB and 1 had RBBB. |
| Virmani 1988(30)  Recruited - 1983-1986 | Autopsy study, Maryland, USA | Autopsy findings of 6810 patients reviewed of which 40 had detectable cocaine, its metabolities, or both in body fluids. Of these, 31 had natural cocaine associated deaths (mean age 28 +/- 5, 22 males, 9 females, blood level of cocaine 0.3 +/- 0.3mg/L) and 9 had homicidal deaths (mean age 33 +/-8, 7 males, 2 females,blood level of cocaine 0.3 +/- 0.3mg/L). 40 cocaine associated deaths were compared to 27 controls with sudden traumatic death ) and no detectable evidence of drug abuse. (mean 34+/- 3; 25 males, 2 females). Ethnicity not stated | Case-control study | N/A. Observational | Forensic pathologists recorded extent of epicardial coronary atherosclerosis in any four major epicardial coronary arteries (right, left main, left anterior descending, left circumflex) by transverse cuts at 5mm intervals.  Severe coronary atherosclerosis was defined as luminal narrowing in cross section of >75%.  Sections reviewed for presence or absence of myocarditis, diagnosed by means of modified Dallas Criteria. Also reviewed for presence of contraction band necrosis (hypereosinophilic bands traversing the myocyte cytoplasm graded according to absent; present focally; or large area of involvement* | Two of the cocaine cases had severe coronary atherosclerosis and one an occlusive coronary thrombus. Numbers of control group with atherosclerosis or coronary thrombus not stated. Histological examination showed myocarditis (mononuclear infiltrate) in 8 of the 40 cases (20%) compared to 1 of the 27 controls (3.7%, p<0.05, CI not stated).  Contraction band necrosis occurred in 25% of cocaine-associated deaths compared to 41% incidence amongst victims of sudden traumatic death. |

*Also evaluated non thrombogenic outcomes of gross coronary morphology (examined for weight; right and left ventricular thickness; valvular abnormalities) results of which are not reported here

† Also evaluated for haemodynamic monitoring (e.g. continuous ECG, systemic arterial pressure, heart rate), results of which are not reported here
